# An Integrated Biomarker Approach Using Flounder to Improve Chemical Risk Assessments in the Heavily Polluted Seine Estuary

**DOI:** 10.3390/jox10020004

**Published:** 2020-10-27

**Authors:** Elodie Borcier, Grégory Charrier, Jérôme Couteau, Géraldine Maillet, Fabienne Le Grand, Antoine Bideau, Matthieu Waeles, Stéphane Le Floch, Rachid Amara, Vianney Pichereau, Jean Laroche

**Affiliations:** 1 Laboratoire des Sciences de l’Environnement Marin, Institut Universitaire Européen de la Mer, Université de Bretagne Occidentale, UMR 6539 CNRS/UBO/IRD/Ifremer, Place Nicolas Copernic, 29280 Plouzané, France; elodie.borcier@univ-brest.fr (E.B.); gregory.charrier@univ-brest.fr (G.C.); fabienne.legrand@univ-brest.fr (F.L.G.); antoine.bideau@univ-brest.fr (A.B.); matthieu.waeles@univ-brest.fr (M.W.); vianney.pichereau@univ-brest.fr (V.P.); 2 TOXEM, 12 rue des 4 saisons, 76290 Montivilliers, France; jerome.couteau@toxem.com (J.C.); geraldine.maillet@toxem.com (G.M.); 3 CEDRE, 715 rue Alain Colas, 29200 Brest, France; stephane.le.floch@cedre.fr; 4 Laboratoire d’Océanologie et Géosciences UMR 8187, Université du Littoral, 28 Avenue du Maréchal Foch, 62930 Wimereux, France; rachid.amara@univ-littoral.fr

**Keywords:** *Platichthys flesus*, estuaries, biomarkers, bioenergetics, polar lipids, population genetics

## Abstract

The objective of this study was to develop an integrative approach in ecotoxicology (from biomarkers to population genetics) to assess the ecological status of fish populations. Flounders (*Platichthys flesus*) collected after the spawning season in the heavily polluted Seine estuary were compared with the moderately polluted Bay of Douarnenez. The muscle energetic reserves were highly depleted in Seine vs. Douarnenez fish. The Seine fish displaying a reduced capacity to manage the oxidative stress and a higher energetic metabolism. An increase in the content of muscle membrane phospholipids (sphingomyelin, phosphatidylserine, free sterols) was detected in the Seine vs. Douarnenez fish. The data integration allowed to hypothesize relationships between membrane phospholipids, xenobiotic metabolism, bioenergetics, and antioxidant defence. The genetic diversity considering neutral markers was maintained in the heavily polluted Seine population compared with the Douarnenez population. Finally, we suggest that the high physiological cost of tolerance to toxicants in the Seine flounder population could compromise its capacity to respond in the future to an additional stressor like warming waters in shallow depth. Thus, this population could be submitted to an ecological risk.

## 1. Introduction

All over the world, estuaries are affected by numerous anthropogenic pressures, e.g., diffuse chemical pollution and dredging inducing an alteration of the water quality, eutrophication increasing the risk of hypoxia, and expansion of harbours, industries, and cities reducing intertidal areas. Furthermore, these shallow estuarine ecosystems are particularly exposed to heat stress related to global climate change, and are thus increasingly subjected to multistress [1,2,3,4,5].

Over European coastal areas and estuaries, the impacts of contaminants are currently assessed using integrated approaches encompassing different matrices (sediment, fish, molluscs) and end-points (chemical analyses, biological effects) [6,7]. The main objective of such approaches is to assess the status of marine environments in the context of the Marine Strategy Framework Directive [8].

The flounder (*Platichthys flesus)* is a major sentinel species to explore the environmental status of European estuarine ecosystems. This weakly exploited flatfish is broadly distributed from Portugal to Norway, with young juveniles being recruited in estuaries where they live for at least two years, before migrating to the mouth of the estuaries for the reproduction, with most individuals returning to their original estuary after spawning [9]. Fitness loss (reduced fish growth and condition index, weak fecundity), modification in gene expression patterns (alterations in apoptosis, energy metabolism and immunity pathways; responses to oxidative stress; induction of detoxification), and “biomarker” signals have been detected in flounder populations located in chronically contaminated environments [10,11,12,13,14,15,16,17].

In natura observations and common garden experiments have shown contrasting patterns of energy metabolism detected by transcriptomic, enzymatic and proteomic approaches [14,18,19] and differential membrane phospholipid composition [20] in European flounder populations. These bioenergetic differences may reflect differential capacities of flounder populations to cope with environmental stressors [20,21]. In addition, the Cytochrome C Oxidase activity (CCO: the terminal electron acceptor in the mitochondrial electron chain transport) and the G6PDH activity (involved in the pentose phosphate pathway shunt) could be considered as pertinent proxies of respectively aerobiosis and defence against oxidative damage [19,20]. Finally, population genetics studies conducted on the European flounder highlighted probable selective pressures acting on various genes involved in detoxification, apoptosis and bioenergetics, thus modifying the genetic variability in polluted vs. moderately contaminated populations [22,23,24].

In the present study we have compared levels of chemical contaminants (heavy metals, PCBs, PAHs) and phenotypic signatures in flounder populations (condition index, biomarker of defense (EROD), biomarker of damage (Acetycholinesterase), bioenergetics, and muscle lipid composition), in flounder populations from two contrasted environments: the moderately contaminated bay of Douarnenez [25] vs. the Seine estuary that is ranked among the most highly polluted European estuaries [16].

We expect that the post-spawning stage could be a sensitive period of the life cycle in polluted environments, since the physiological cost of reproduction could lead to decreased stress resistance [26,27]. Thus, adult flounders were collected after the spawning season, spent fish being characterized by the lowest condition index and muscle lipid content [28].

Population-genetic methods applied to wild fish exposed to pollution may improve our understanding of demographic process [29,30]. Since the life cycle of *P. flesus* could promote the development of fine-scale population structure, we have compared the genetic diversity of Douarnenez vs. Seine populations, considering neutral markers (microsatellites), to assess the level of genetic differentiation between them and explore a possible loss in genetic variability in the heavily polluted population.

Finally, the main objective of the present paper was to develop an integrative approach in ecotoxicology, considering chemical signatures, biomarkers, bioenergetics, and population genetics: (1) to compare the ecological status of fish populations submitted to contrasted levels of pollution in coastal ecosystems, and (2) to assess the ecological risk of a fish population living in one of the most polluted European estuaries, the Seine estuary.

## 2. Materials and Methods

### 2.1. Fish Sampling

**Figure 1:**
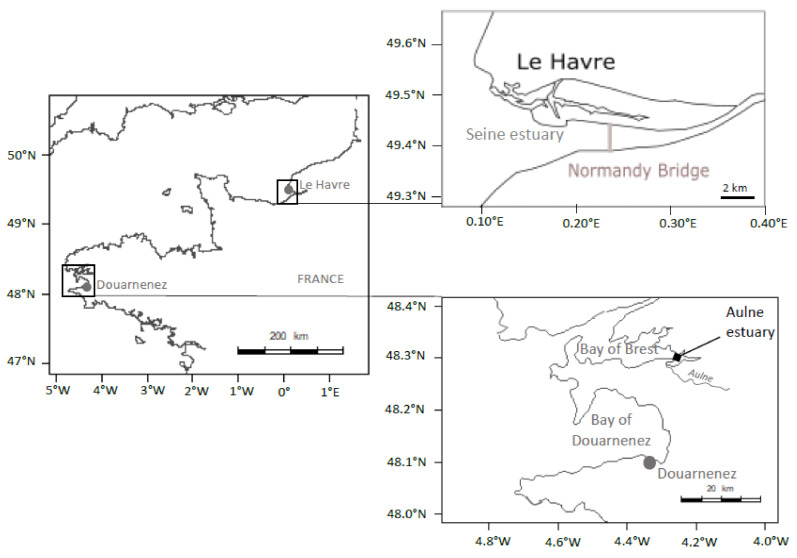
Location of sampling sites (Seine Estuary, Bay of Douarnenez).

Adult flounders (total fish length > 25 cm) were sampled with a beam trawl, over the same period (8–12 March 2016), in the bay of Douarnenez and in the lower part of the Seine estuary, downstream the “Pont de Normandie” (Figure 1). The total river basin of the bay of Douarnenez is characterized by a limited area (230 km^2^), two main rivers (Kerharo and Ris; flow < 3 m^3^/s), a limited human population (29,000 inhabitants) and many agricultural activities [31]. Levels of metals (Cd, Pb, Hg, Cu, Zn), PAHs (fluoranthene and phenanthrene), PCBs (CB 153, CB 118) in sediment and mussels in the bay of Douarnenez are below the median observed in the northern part of the Bay of Biscay and Brittany [25]. Thus, the bay of Douarnenez could be considered as a moderately contaminated ecosystem.

The Seine river basin, on the other hand, is characterized by important cities (Paris: 10 million inhabitants, Rouen: 400,000 i., Le Havre: 250,000 i.), large harbours (Rouen, Le Havre) and major industrial areas (Elbeuf, Rouen, Le Havre). The average annual Seine flow measured 150 km upstream from the mouth of the estuary is relatively high (500 m^3^/s; [32]). Despite a general improvement of the Seine water quality since 1990, levels of Pb, Zn, Cd, and Hg in sediments and levels of Ag, Pb, and Cd in mussels remain very high relatively to the median levels detected over the French Atlantic coasts. Furthermore, levels of PAHs in mussels are higher compared to the median contamination of the Atlantic coast, while levels of PCBs in sediments and mussels are clearly the highest of the French coasts [32]. Thus, the Seine estuary can be considered as a heavily polluted ecosystem.

Thirty six fish were collected per estuary and euthanized by cervical dislocation immediately after capture. For each individual sample, sex and maturation stage were determined macroscopically, and the total length and gutted carcass weight were measured. The whole brain, the liver and a sample of muscle were snap-frozen and stored in liquid nitrogen until further analysis. Two pools of five muscle samples and five liver samples were also collected in each estuary, and preserved at −20 °C for chemical analyses (metals, PCBs, PBDEs, PAHs). A caudal fin fragment was collected individually and stored in 95% ethanol for genetic analysis.

The Fulton’s condition index (K) was assessed with the formula: K = 100 × (W/L^3^) where W = gutted body weight (i.e., carcass without internal organs) (g) and L = total length (cm) [33]. The hepato-somatic index (HSI) was calculated with the equation: HSI = 100 × (LW/W) where LW = liver weight (g) [34].

### 2.2. Metal Analysis

After weighing 200 mg of dried and powdered material, digestions were performed at 105 °C for 4 h in closed 15-mL Teflon screw-cap vials (Savillex, Minnetonka, MN, USA) with 4 mL suprapur 65% nitric acid (Merck, Darmstadt, Germany) and 1 mL suprapur 30% hydrogen peroxide (Merck, Darmstadt, Germany). Measurements of metals were conducted on diluted mixtures (2.5% HNO_3_) using an ICP-quadrupole mass spectrometer (X-series II, Thermo Scientific) operated at the Pole Spectrometry Ocean Brest (PSO, Brest, France). All concentrations shown in the present study were well above detection limits while digestion blanks were below detection limits. Three certified reference materials from NRCC (National Research Council of Canada, Ottawa, ON, Canada), including fish protein, dogfish liver, and lobster hepatopancreas, were used for assessment of the method accuracy (Table 1).

### 2.3. Organic Pollutants Analysis

The concentration of 24 PAHs, 26 PCBs, 28 pesticides and 7 PBDEs was assessed in fish tissues by stir bar sorptive extraction-thermal desorption-gas chromatography-tandem mass spectrometry (SBSE-GC-MS/MS) using a method adapted from [35]. Briefly, for each organism, 100 mg wet weight (w.w.) of tissue was digested by saponification and analytes were extracted by stirring during 16 h at 700 rpm using polydimethylsiloxane stir-bars (Twister 20 mm × 0.5 mm, Gerstel). Bars were subsequently analysed using a gas chromatography system Agilent 7890A coupled to an Agilent 7000 triple quadripole mass spectrometer (Agilent Technologies) and equipped with a Thermal Desorption Unit (TDU) combined with a Cooled Injection System (Gerstel). The GC column was a Restek Rxi-5ms (30 m, 0.25 mm, 0.25 µm). Analytes were quantified relatively to deuterated compounds using a calibration curve ranging from 0.01 ng to 30 ng per bar. A mean tissue water percentage of 81% was measured by drying samples at 50 °C until the mass remained constant. Results were expressed as µg analytes/kg dry weight (d.w.). Limits of quantification (LOQ) were calculated by the calibration curve method [36] and limit of detection (LOD) were estimated by dividing LOQ by 3. Analytical quality control was performed using the Standard Reference Materials 1974c “Organics in Mussel Tissue *(Mytilus edulis)*” provided by the National Institute of Standards (SRM) and Technology (NIST, Gaithersburg, USA).

### 2.4. Enzymatic Activities

#### 2.4.1. Glucose 6-Phosphate Dehydrogenase (G6PDH) & Cytochrome C Oxidase (CCO)

Protein extraction was carried out with approximately 100 mg of tissue adjusted to 1/40 for liver tissue and 1/50 for muscle tissue with the corresponding volume of HEPES-EDTA extraction buffer (pH 7.5, 20 mM HEPES (4-(2-hydroxyethyl)-piperazine-1-ethanesulfonic acid), 1 mM EDTA, 0.1% Triton X-100). Liver and muscle tissues were crushed in a mixer mill (MM400, RETSCH, Germany) under nitrogen atmosphere. Tissues were homogenized in ice-cold extraction buffer for three bursts of 20 s using Ultra Turrax T25 tissue homogeniser.

Enzymatic activities were quantified with a 96-well microplate spectrophotometer (OMEGA PolarStar, BMG, Labtech) using 8 × 12 multicell blocks, maintained at a temperature of 22 °C. Reaction conditions varied according to enzymes:

- Glucose 6-phosphate dehydrogenase (G6PDH; EC 1.1.1.49, Gauthier et al. 2008): Imidazole buffer ((imidazole 50 mM, MgCl2 25 mM, KCl 70 mM), pH 7.5, β-nicotinamide adenine dinucleotide phosphate (NADP) 0.3 mM, glucose-6-phosphate 200 mM (omitted in controls). G6PDH activity was undetected in muscle samples and was only measured in liver at 340 nm.
- Cytochrome C Oxidase (CCO; EC 1.9.3.1, [13,20,37]): Na Phosphate Buffer 0.33 M, pH = 7, Cytochrome C 2 mM, DTT 0.1 M. Activity of CCO was measured at 550 nm.

To calculate the protein concentration, the Coomassie blue method (Bradford, 1976) was applied using bovine serum albumin as a standard (BIORAD Laboratories, USA): 10µL of the dilution (1/40 or 1/50) were put in 200 µL of Bradford reagent, following a dilution range (from 0.5 mg.mL^−1^ to 0.0625 mg.mL^−1^ for liver and from 0.5 mg.mL^−1^ to 0.031 mg.mL^−1^ for muscle). The absorbance DO was read at 595 nm. CCO and G6PDH activities were expressed in international unit (IU) per milligram of protein.

#### 2.4.2. Ethoxyresorufin-O-Deethylase (EROD) & Acetylcholinesterase (AChE)

The liver and brain were weighed and homogenized in cold 0.1 M phosphate buffer pH 7.8 (and 0.1% Triton 100X for brain) (250 mg brain tissue per ml of buffer), and extractions were performed with a manual homogenizer. Homogenates were centrifuged at 4 °C for 20 min at 10,000× *g* and supernatants were collected for measuring EROD and cholinesterase activities and performing protein assays.

Liver ethoxyresorufin-O-deethylase (EROD) activity was measured in quadruplicate as described by [38]. The reaction volume contained 200 µL phosphate buffer (pH 7.8, 0.1 M), 10 µL ethoxyresorufin (46 µM), and 10 µL of aliquots of supernatants. The reaction was initiated by addition of 10 µL of NADPH (10 mM) to the reaction volume. The progressive increase in fluorescence, resulting from resorufin formation, was measured during 8 min (excitation wavelength 530 nm, emission wavelength 585 nm).

Brain AChE activity was determined within 24 h in quadruplicate according to the colorimetric method of [39] at room temperature. Acetylthiocholine iodide (ATCh) was used with 5,5-dithiobis-2-nitrobenzoate (DTNB) as the thiol indicator. The reaction volume contained: 340 µL phosphate buffer (pH 7.8, 0.1 M), 20 µL DTNB (0.01 M in 0.1 M phosphate buffer, pH 7.8) and 10 µL of aliquots of supernatants. The reaction was initiated by addition of 10 µL ATCh 0.1 M to the reaction volume. The rate of TNB production was evaluated during 2 min at 412 nm to estimate substrate hydrolysis.

Proteins were quantified by the Coomassie blue method [40] using bovine serum albumin as standard. AChE and EROD activities were respectively expressed as μmol of acetylthiocholine (ATCh) hydrolyzed per minute per milligram of protein and pmol of resorufin per minute per milligram of protein.

### 2.5. Lipids Analysis

Muscles were frozen in liquid nitrogen and ground into powder. Lipids were extracted from 150 mg of muscle powder in 6 mL of chloroform:methanol (2:1, v:v) [41]. Neutral and polar lipid classes were analysed by high-performance thin-layer chromatography (HPTLC) following a method adapted for *P. flesus* [20] and using a CAMAG HPTLC complete system (autosampler and scanner). Six neutral lipid classes (sterol esters (STEST), glyceride ethers (GLETH), triacylglycerols (TG), free fatty acids (FFA), fatty alcohols (ALC), and free sterols (FST)) and seven polar lipid classes (sphingomyelin (SPG), lysophosphatidylcholine (LPC), phosphatidylcholine (PC), phosphatidylserine (PS), phosphatidylinositol (PI), cardiolipin (CL) and phosphatidylethanolamine (PE)) were identified by standard comparison (Sigma Aldrich) and quantified relatively to standard calibration curves (Visioncats software, CAMAG). Neutral lipids, except FST, are storage lipids, whereas polar lipids and FST are membrane lipids. Data were expressed as µg of lipid per milligram of fresh weight. The lipid storage index (TG/FST) based on the ratio of the quantity of reserve lipid (TG) to the quantity of structural lipid (free sterols: FST), classically considered in fish population studies [42], was assessed.

### 2.6. Genetic Diversity

#### 2.6.1. Genotyping 

DNA extraction was performed according to a phenol chloroform protocol adapted from [43]. DNA concentration was estimated using a NanoDrop 8000 spectrophotometer (ThermoFisher Scientific) and DNA samples were diluted to 20 ng^.^mL^−1^ in ultrapure water for molecular biology, DNase, RNase and protease free (EMD Millipore).

A set of 20 microsatellites was successfully amplified and optimized [24,44,45,46,47,48]. Each locus was amplified by simplex polymerase chain reaction (PCR) in 10 µL volume (5X green Go Taq reaction buffer (Promega), 0.5–1.5 mM MgCl_2_, 0.2 mM each dNTP, 0.02 mM forward primer, 0.2 mM reverse primer, 0.2 mM universal primer (fluorescent), 0.25 U green Go Taq polymerase (Promega), 1 µL DNA template). PCR amplifications were performed in a Geneamp PCR System 9700 (Applied Biosystems). A touchdown procedure was included in the thermal cycling regime.

The 20 microsatellites were grouped into three panels. For each panel, 3 µL of each amplified locus (except 6 µL for Nplaf_28) were mixed together. One µL of pooled PCR products was mixed with 10µL Hi-Di formamide and 0.15 µL GeneScan 500-LIZ size standard (Applied Biosystems). The sequencing of PCR products was carried out on a capillary sequencer ABI 3130 (Applied Biosystems). Electrophoregrams were analysed with GeneMapper v.4.0 (Applied Biosystems), and were scored independently by two readers in order to minimize genotyping errors. Individuals with more than 50% missing data were removed from the data set, resulting in 30 individuals successfully genotyped per population (Douarnenez and Seine). For downstream analyses, the genotype matrix was converted into proper input files with CREATE 1.37 [49].

#### 2.6.2. Analysis of Genetic Diversity

Allelic diversity (N_a_) was calculated with GENETIX 4.05 [50]. Observed and expected heterozygosities (H_o_ and H_e_, respectively) were assessed for each locus and over all loci in each population using GENETIX 4.05 [50]. F_IS_ were estimated for each locus and population in GENETIX in order to evaluate departures from Hardy–Weinberg equilibrium (HWE), and their significance was tested with 5000 permutations. The occurrence of null alleles and genotyping errors was verified with MICRO-CHECKER v.2.2.3 [51]. In order to evaluate the population genetic differentiation, F_ST_ was estimated with GENETIX v 4.05 [50], and its significance was tested with 5000 permutations.

### 2.7. Statistical Analyses

Statistical analyses were performed with R software (v.3.5.0) implemented in Rstudio (v. 1.1.453). Differences between populations were tested using t-test when the homoscedasticity and homogeneity of variances were respected. Otherwise, the Kruskal–Wallis test was applied. Graphical analyses were performed with “ggplot” package of R. A *p*-value lower than 0.05 was considered as a significant difference. Principal component analyses were performed with the FactorMineR [52] package with default settings.

## 3. Results

### 3.1. Contaminant Concentrations

**Figure 2:**
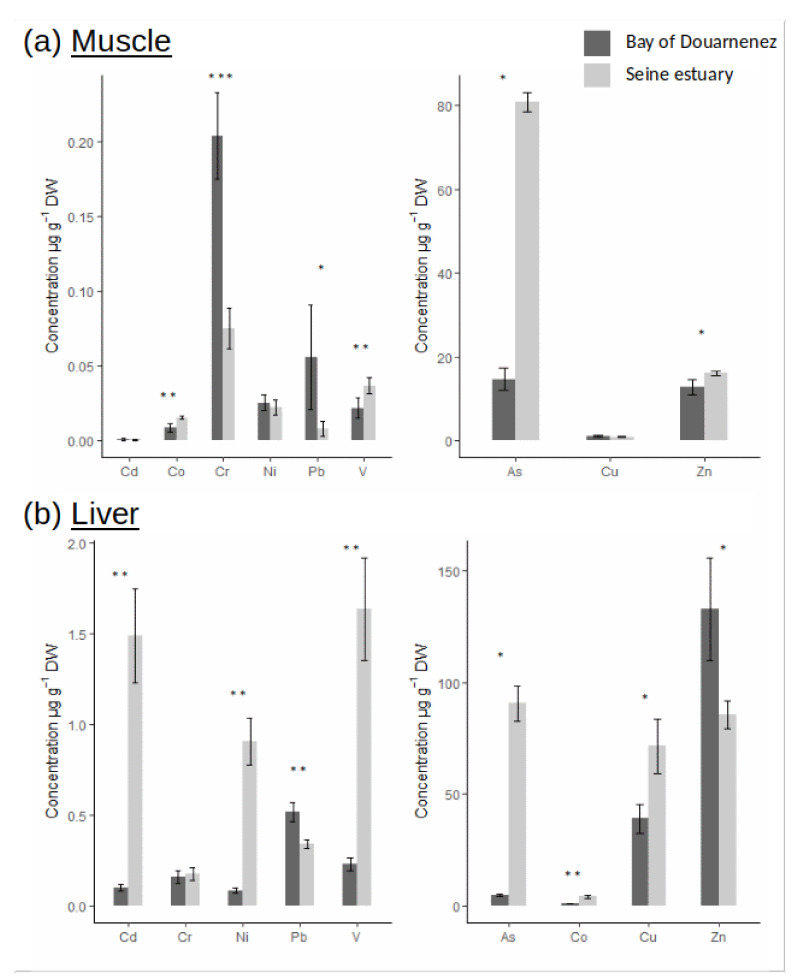
Metal concentrations (µg.g^−1^ DW) in muscle (**a**) and liver (**b**), for two fish populations (Seine and Douarnenez). (*p*-value following Kruskal-Wallis test: *: *p*-value < 0.05; **: *p*-value < 0.01; ***: *p*-value < 0.001).

The bioaccumulation of as in muscle was much higher in Seine vs. Douarnenez (ratio = 5.5), and the concentration of particular metals (Co, V, Zn) in muscle was also significantly higher in Seine (ratio ≈ 1.5) (Figure 2a). On the other hand, higher muscle concentrations of Cr and Pb were detected in Douarnenez vs. Seine (5.5 < ratio < 7). No differences in bioaccumulation of Cd, Ni and Cu in muscle was observed between the two flounder populations (Figure 2a). Levels of metals were two to ten times higher in liver vs. muscle (Figure 2b), and patterns of metal concentration in liver were very different from those observed in muscle. Concentrations of Cd, Ni, V, and As in liver were significantly higher in Seine vs. Douarnenez (7 < ratio < 20). An inverse trend was detected for Pb and Zn concentrations in liver which were higher in Douarnenez vs. Seine (ratio = 1.5) (Figure 2b).

The analysis of organic pollutants (PAHs and PCBs) in flounder tissues confirmed the differential contamination status of Seine vs. Douarnenez. The PAH naphthalene was detected in liver in Seine (97 ng.g^−1^ DW), but not in Douarnenez (Table 2). Furthermore, the concentrations of PCBs in liver (101, 118, 153, 138, 180) were higher in Seine (i.e., PCB 153: 255 ng.g^−1^ DW in Seine vs. not detected in Douarnenez; PCB 138: 290 ng.g^−1^ DW in Seine vs. 45 ng.g^−1^ DW in Douarnenez). The same trend was observed in muscle, with PCBs (101, 118, 153, 138) having concentrations varying from 69 to 167 ng.g^−1^ DW in Seine fish, while being not detected in Douarnenez fish (Table 2).

### 3.2. Fish Biometry, Condition Index, Hepato-Somatic Index, Muscle Protein Concentration

The sex ratio was unbalanced in the two populations and showed a majority of females in Seine (sr = m/f = 0.38) vs. a majority of males in Douarnenez (sr = 6.20). Average fish lengths (± SD) were not significantly different in Seine (31.93 ± 1.87 cm) compared with Douarnenez (33.10 ± 4.45 cm). The condition factor k (mean ± SD) was significantly lower in Seine (0.74 ± 0.07) vs. Douarnenez (0.95 ± 0.11) (Kruskal–Wallis, *p* < 0.001), but the hepato-somatic index HSI was not significantly different in Seine (1.37 ± 0.26) vs. Douarnenez (1.45 ±.0.53).

The muscle protein level (mean ± SD), was significantly lower in Seine (34.15 ± 8.36 mg.ml^−1^) vs. Douarnenez (42.21 ± 7.33 mg.ml^−1^) (Kruskal–Wallis, *p*-value < 0.001).

### 3.3. G6PDH, CCO, AChE and EROD Activities

**Figure 3:**
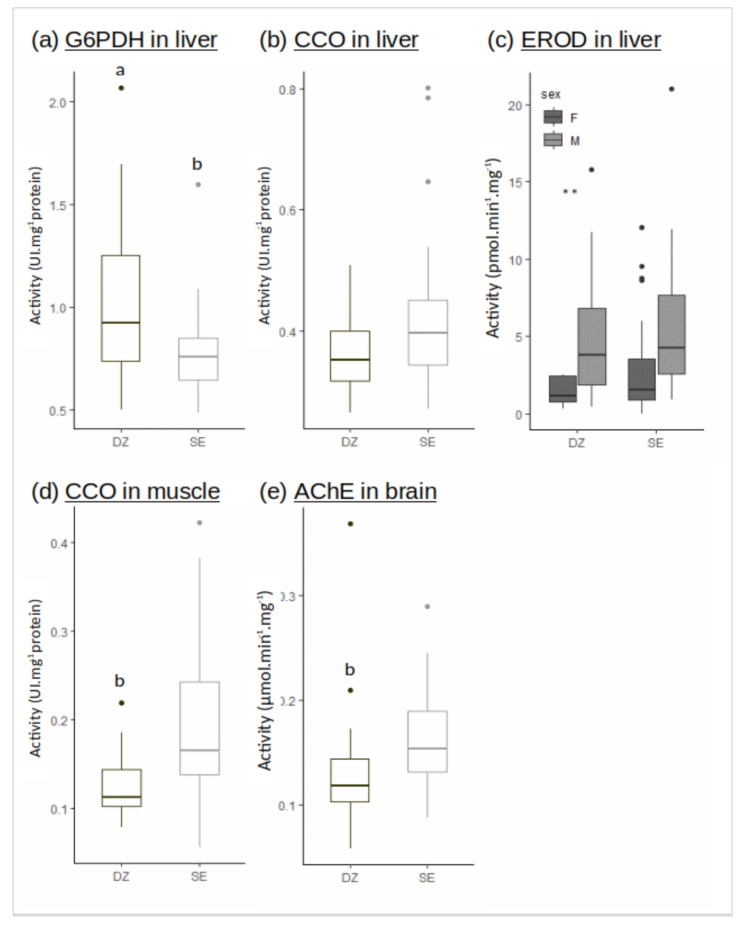
Enzymatic activities in liver ((**a**) G6PDH, (**b**) CCO, (**c**) EROD), muscle ((**d**) CCO), brain ((**e**) AChE) in the Douarnenez bay (DZ) and in the Seine estuary (SE). Letters indicate significant difference between mean flounder population of DZ and SE (Kruskal-Wallis test: *p*-value < 0.001). Two stars ** indicate a significant difference within the Douarnenez population (Kruskal-Wallis test: *p*-value < 0.01).

The G6PDH activity in liver was significantly reduced in Seine vs. Douarnenez (Figure 3a). The CCO activity in liver was not different between Seine and Douarnenez, while the CCO activity in muscle was higher in Seine (Figure 3b,d).

The AChE activity in brain was significantly reduced in Douarnenez vs. Seine (Figure 3e). The EROD activity in liver was measured considering sexes separately, a general decrease of the activity being observed in females vs. males over the two populations. No significant difference was recorded in EROD activities when individuals belonging to the same sex were compared between the two flounder populations (Figure 3c).

### 3.4. Muscle Lipids

The level of total lipids in muscle was significantly reduced in Seine (9.88 µg.mg^−1^) vs. Douarnenez (12.21 µg.mg^−1^) (Table 3). In the two populations, triacylglycerols (TG) were the major reserve lipid whereas phosphatidylcholine (PC), phosphatidylinositol (PI) and phosphatidylethanolamine (PE) were the main membrane lipid classes. A significant decrease of the lipid storage index (TG/FST ratio) was observed in Seine (2.38 ± 3.15) vs. Douarnenez (5.06 ± 4.65) (Kruskal–Wallis, *p*-value < 0.01).

The majority of membrane lipids (LPC, PC, PI, CL, and PE) displayed higher levels in Douarnenez vs. Seine; an opposite trend being detected for the group (FST, SPG, PS: free sterols, sphingomyelin, phosphatidylserine) which showed higher concentrations in Seine vs. Douarnenez (Table 3).

**Figure 4:**
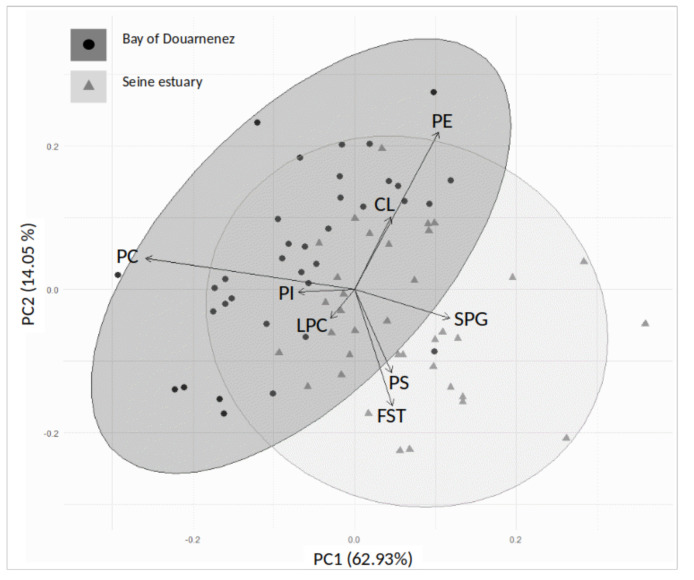
Principal component analysis (axes 1 and 2). Distribution of membrane phospholipids on the correlation circle, and individuals on the factorial plan, in the Douarnenez and Seine populations. SPG: sphingomyelin, LPC: lysophosphatidylcholine, PC: phosphatidylcholine, PS: phosphatidylserine, PI: phosphatidylinositol, CL: cardiolipins, PE: phosphatidylethanolamine, FST: free sterols.

The distribution of membrane lipids and individuals was analysed in the two estuaries by a principal component analysis (PCA), with the main factorial plan (axes 1 and 2) explaining 76.98% of the total variance of the data set (Figure 4). The Seine fish were mainly segregated from those of Douarnenez by higher concentrations of FST-PS-SPG, lower concentrations of PC, and less variable levels of PE (Figure 4).

### 3.5. Integration of the Phenotypic Responses

Two principal component analyses were performed successively for the two flounder populations, to integrate condition factor (K), muscle protein, enzymatic activities (liver G6PDH, muscle CCO, brain AChE, liver EROD) and the membrane lipids displaying higher concentrations in the heavily polluted Seine system (SPG, FST, PS).

**Figure 5:**
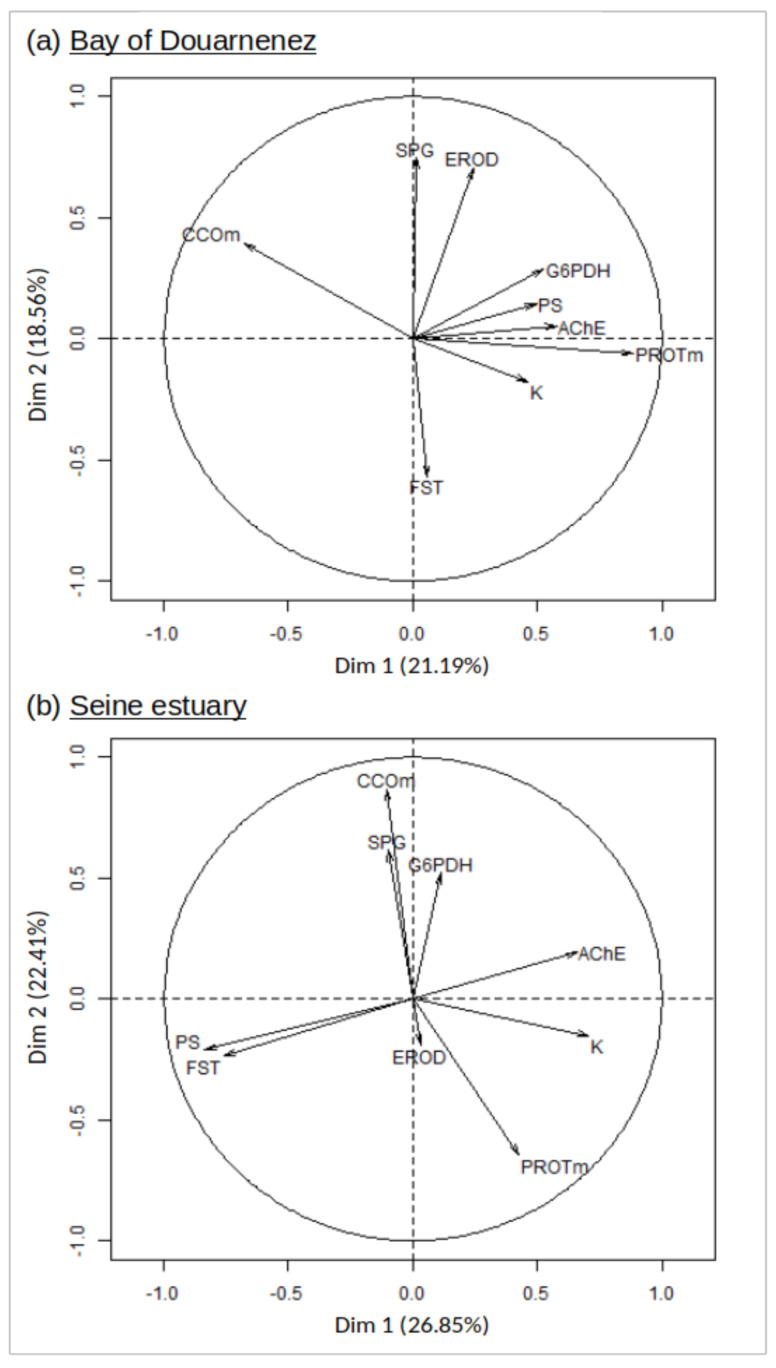
Principal component analysis (axes 1 and 2). Distribution of 9 markers on the correlation circle, in the Douarnenez and Seine populations. G6PDH: liver glucose-6-phosphate deshydrogenase, CCOm: muscle Cytochrome C Oxydase, EROD: liver e*thoxyresorufine-O-dééthylase,* AChE: brain acetylcholine esterase, PROTm: muscle protein concentration, SPG: sphingomyelin, FST: free sterols, PS: phosphatidylserine, K: condition factor.

The two first axes of the PCA explained 44.75% and 49.26% of the total variance of the data set, respectively for Douarnenez and Seine populations (Figure 5). In the Douarnenez sample, a group of positively correlated variables (K-muscle protein-G6PDH-AChE-PS) was negatively correlated with CCO on the first axis, with the second axis showing a negative correlation between EROD-SPG and FST (Figure 5).

In the Seine sample, a group of positively correlated variables (K-AChE) was negatively correlated with the group (PS-FST) on the first axis. The second axis displayed a group of positively correlated variables (G6PDH-CCO-SPG) which was negatively correlated with muscle protein (Figure 5).

### 3.6. Genetic Variability

The genetic diversity was variable among microsatellites, with the total number of alleles per locus ranging from 3 to 21, and the mean observed heterozygosity per locus varying between 0.166 and 0.971 (Table 4). Over all loci, the mean allelic diversity (Na) was not statistically different in Douarnenez vs. Seine populations (respectively 7.45 and 6.8 alleles per locus). The mean observed heterozygosity was also similar in the two populations (H_o_ = 0.56) (Table 4). No significant deviation from Hardy–Weinberg equilibrium (HWE) was detected, regardless of the considered population (multilocus F_is_ = 0). The software MICRO-CHECKER did not detect any significant departure from HWE linked to null alleles. No significant genetic differentiation was detected between the two populations (multilocus F_st_ = 0.003, *p* >0.05).

## 4. Discussion

### 4.1. Chemical Contamination & Hypothesis on the Flounder Use of Habitat in the Seine Estuary and Douarnenez Bay

Data on the level of contaminants in the sediments of our two sampling sites are available [25,32]. They highlighted significant higher levels of heavy metals and PAHs in the sediments of Seine vs. Douarnenez, expressed as µg g^−1^ dry wt. (respectively Cd: 1.4/0.1; Pb: 60/20; Hg: 0.54/0.02; Zn: 160/80; major PAHs: 1/0.1).

In the present paper, the Cd concentrations in muscle were moderate comparatively to the heavily polluted Gulf of Gdansk in southern Baltic Sea [17] (flounder muscle: Cd ≅ 0.004 µg g^−1^ dry wt.), and the levels of Pb and Zn were rather similar to those in the Baltic Sea (flounder muscle: Pb ≅ 0.04; Zn ≅ 20 µg g^−1^ dry wt.).

The fish liver is often recommended as a target tissue when monitoring metal concentrations in aquatic environments [53]. Accordingly, in the present study, higher concentrations were classically detected in liver for the majority of metals, the ratio liver vs. muscle concentrations being comprised between 3 and 99. Two exceptions were detected for Cr and As which displayed similar concentrations in liver and muscle. Levels of Cd, Ni, V and Cu (µg·g^−1^ dry wt.) in liver were significantly higher in Seine vs. Douarnenez (respectively: 1.48/0.099; 0.91/0.08; 1.63/0.23; 71.41/38.92). Levels of Cd and Cu in Seine were similar to those observed in the liver of flounder coming from large industrial estuaries in France (Gironde and Loire; [54]). The level of Ni in Seine being similar to those observed in heavily polluted estuaries in England (Tyne and Mersey; [55]).

An inverse trend was detected in the present study, for the Pb and Zn concentrations in liver (µg g^−1^ dry wt) which showed higher values in Douarnenez vs. Seine (respectively: 0.52/0.34; 132/85). The Pb level in liver in Douarnenez could be considered as a high concentration for a moderately polluted system of the French Atlantic coast; coastal zones and small estuaries along the Eastern English Channel and in the Bay of Biscay generally showing reduced Pb levels in flounder liver (0.04 to 0.26; [53,54]).

Levels of organic pollutants detected in flounder liver and muscle highlighted a clear differentiation between the moderately contaminated Bay of Douarnenez and the heavily polluted Seine estuary. Thus, a significant level of a particular PAH (naphthalene) was measured in the Seine flounder liver (97 ng g^−1^ dry wt.), this molecule being not detected in the Douarnenez sample. The naphthalene level detected in the Seine estuary could be associated with a relatively high pollution by PAHs, the concentration of total PAHs in the flounder liver along the French Atlantic coast varying from 10 to 100 ng g^−1^ dry wt. [56].

The level of liver PCBs was considerably higher in Seine vs. Douarnenez, the sum of the seven major PCBs (CB-28, -52, -101, -118, -138, -153, -180) being respectively for these two samples: ∑_7_PCB = 720 vs. 70 ng g^−1^ dry wt. These levels of PCBs in the Seine estuary can be considered as high values, similar to those observed in England polluted estuaries like Tyne and Mersey (∑_7_PCB in liver flounder varying from 400 to 1600 ng g^−1^ dry wt.; [55]). Furthermore, the sum of the major PCB detected in Seine flounder muscle (472 ng g^−1^ dry wt) was considerably higher than those observed in Baltic Sea polluted sites (15 < ∑_7_PCB < 34 ng g^−1^ dry wt.; [17]).

Finally, considering the whole results on the flounder contamination data in the present study, we confirmed that despite a general improvement of the Seine water quality since 1990 [32], the Seine estuary is still highly contaminated by metals and organic pollutants (PAHs & PCBs). The flounder *Platichthys flesus* is realizing the major part of its life cycle (from juvenile stage to adult) in the Seine estuary [32], with all the major habitats for the fish development being available in this large system (nursery areas, feeding habitats, spawning areas). Thus we suggest that this fish is a relevant sentinel species for monitoring the water quality of the Seine system.

In the Bay of Douarnenez, the flounder contamination appeared relatively limited considering organic pollutants and metals, with a notable exception for two metals; concentrations of Pb and Zn in liver being higher in Douarnenez vs. Seine. However, levels of PAHs, PCBs, and metals (including Pb and Zn) in sediment and mussels from the Douarnenez bay were clearly below the median concentrations detected over the French Atlantic Coast [25]. Thus, we suggest that the difference between environmental and flounder contaminations could be linked to the particular status of the Bay of Douarnenez. First mature flounders arrive in the Bay of Douarnenez at the beginning of the spawning season in January. The spawning peak is observed in February, then fish leave the area in March [57]. No significant estuarine systems are available in the Bay of Douarnenez [31], thus the flounder must spend the major part of its life cycle outside the Bay (recruitement and growth of juveniles in estuaries, come back of the adults to the estuary after the spawning period at sea). Déniel [57] suggested that the flounder population could migrate between two main habitats, the Aulne estuary in the Bay of Brest (outside the reproduction period) and the Bay of Douarnenez (spawning period) (Figure 1). In the present study, we suggest that the Pb and Zn signatures detected in flounder from Douarnenez could be linked to the fish contamination in the Aulne estuary, outside the spawning period, with this estuary being characterized by (1) high loads of Pb, Zn, and Ag in sediments and oysters linked to ancient mining activities, and (2) limited levels of organic pollutants [25].

### 4.2. Condition Index, Muscle Protein Content, Antioxidant Defences and Bioenergetics

The spawning peak was observed in February for *Platichthys flesus* in the Eastern English Channel [58]. Thus, adult fish collected in Douarnenez and Seine in March 2016 were in post-spawning stage that is characterized by a general decrease of the fish reserves due to the cost of reproduction [28]. In the present study, a significant reduction of the condition index (−23%) and muscle protein content (−20%) underlined a fitness loss in the heavily polluted Seine population compared with the moderately contaminated Douarnenez population.

The liver is an essential metabolic organ [59] that shows high production of reactive oxygen species (ROS: by-products of oxygen metabolism) that should be counterbalanced by powerful protective mechanisms to detoxify and repair damaged lipids and proteins [60]. G6PDH is the main source of the reducing power NADPH for biotransformation and detoxification reactions for *P. flesus* [61] and is also involved in biosynthetic processes such as fatty acid synthesis [62]. Prolonged challenges of the flounder liver by pollutants in highly polluted estuaries in the North Sea inhibit the G6PDH activity; as a result, the lower availability of NADPH that is required for detoxification reactions conducts to liver pathology [61,63]. Pollution induces oxidative stress in aquatic organisms [60] since many pollutants are redox cycling compounds and can induce lipid peroxidation and protein oxidation (carbonylation). Antioxidant enzymes themselves are sensitive to damage by ROS, and G6PDH itself may be inactivated by pollutants due to its carbonylation [60,64]. Thus, in the present study, the reduced G6PDH activity in flounder liver in Seine could be related to a lower capacity of this population to respond to oxidative stress in a highly polluted environment and thus to a fitness loss for the Seine population.

Animals use oxygen primarily for energy generation via oxidative phosphorylation, and 90% of oxygen consumed by an organism is used for the reduction of oxygen molecules to water by Cytochrome C Oxidase (CCO); in particular, the CCO activity in muscle is considered as a proxy of fish maximum metabolic rate [65]. Previous studies conducted on flounder juveniles underlined the relevance of the CCO activity in muscle to detect a higher energy metabolism in polluted vs. pristine populations [13] or to identify contrasted patterns of energy metabolism in northern vs. southern populations [20]. In the present study, a significant increase of the muscle CCO activity was detected in Seine vs. Douarnenez flounders, suggesting a higher energy metabolism in the heavily polluted population. This enhanced metabolism was also observed in a pollutant resistant *Fundulus heteroclitus* population in a North American estuary and could be heritable [66]. Thus, we suggest that the higher energy metabolism in the highly polluted Seine flounder population could be an adaptation to cope with the elevated costs of basal metabolism and activation of mechanisms for protection and damage repair.

### 4.3. Neurotoxicity and Xenobiotic Metabolism

In the present study, the brain AChE activity was significantly lower in Douarnenez vs. Seine, these activities being close to those observed in flounders collected in the Minho estuary (Portugal) in November (130 to 174 nmol/min/mg protein; [15]). The acetylcholinesterase (AChE) activity is commonly used as a biomarker of neurotoxicity; its inhibition has been studied during exposure to organophosphorus and carbamate used as active agents in pesticides [67]. This biomarker is also sensitive to others compounds including metals, detergents or hydrocarbons [68,69,70]. Heavy metals in the field [71] and experimental contaminations of zinc and lead have been shown to inhibit fish AChE activity [72,73]. Therefore, we suggest that the reduced flounder AChE activity observed in Douarnenez vs. Seine populations could be mainly linked to a high zinc contamination.

Induction of cytochrome P450 1A (CYP1A) is a widely used biomarker for the study of pollution in aquatic ecosystems, with the activity of 7-ethoxyresorufin-O-deethylase (EROD) being a common method to assess the activity of the CYP1A isoenzyme, playing a role in the metabolism of a large number of compounds such as PCBs and PAHs [74]. In the present work, a higher EROD activity was observed in males vs. females in the two populations, with this difference being linked to the suppressive effect of the hormone 17 β-estradiol produced by female fish during the gonadal maturation process [75]. Furthermore the EROD activity was relatively similar in Seine and Douarnenez, whatever the sex. Levels of EROD activity in both regions were very close to those observed in juvenile flounders sampled in the Minho estuary in the North of Portugal (1.6 < EROD < 5.82 pmol/min/mg protein) that is weakly impacted by organic contaminants [15]. The low EROD values in Seine detected in the present study as in a recent past (flounder EROD in 2009 = 4.2 pmol/min/mg protein; [16]) are surprising, this estuary being characterized by high levels of organic pollutants. We suggest that in the polluted Seine estuary, the flounder population may have developed a resistance to organic pollutants and thus a reduced inducibility of the enzyme cytochrome P450 1A. This resistance could be a plastic response and/or a genetic adaptation, as observed in fish inhabiting chronically contaminated rivers [76]. On the other hand, we suggest that in polluted but open coastal zones in the southern Baltic Sea, the possibility to develop a local adaptation to organic pollutants is more limited for flounder populations which consequently displayed very high levels of EROD activity (from 231 to 279 pmol/min/mg protein; [17]). In the southern Baltic Sea, we hypothesize that a high gene flow from neighbouring populations less exposed to contamination could be a major factor masking the selective pressure [77].

### 4.4. Storage Lipids and Membrane Lipids in Muscle

The triacylglycerol (TG) is the main form of stored energy for fish, free sterols (FST) and phospholipids playing a structural role in cell membranes. The lipid storage index TG/FST was developed to study the nutritional status of fish and was generally seriously depleted in polluted conditions [42,78]. This ratio was reduced by 50% in the Seine estuary relative to moderately polluted Douarnenez fish, and confirmed that the flounder nutritional status is poor in the heavily contaminated system.

Levels of membrane lipids underlined a significant increase of particular molecules in Seine vs. Douarnenez fish, namely free sterols (FST), sphingomyelin (SPG), and phosphatidylserine (PS), and a significant decrease of phosphatidylcholine (PC) and phosphatidylinositol (PI). Furthermore, the interindividual variability of the levels of cardiolipins (CL) and phosphatidylethanolamine (PE) was higher in Douarnenez vs. Seine flounder populations.

Numerous studies have shown that aging and decreased lifespan are associated with a high production of reactive oxygen species by mitochondria, increased mitochondrial DNA and protein damage, and with changes in the fatty acid composition of the mitochondrial membrane [79]. For example, CL are phospholipids of unusual structure, localized almost exclusively within the inner mitochondrial membrane, and are particularly rich in unsaturated fatty acids. CL are thus very susceptible to attack by oxygen radicals and other organic radicals [80]; lipid peroxidation should not be perceived solely in a “damage to lipids” scenario, but should also be considered as an endogenous source of damage to proteins and DNA. On the other hand, the long saturated fatty acid chains of SPG particularly located in the endoplasmic reticulum membrane facilitate their association with cholesterol to form heterogeneities in the membrane that are called lipid microdomains or rafts [81]. An important proportion of Cytochrome P450s are localized in lipid rafts which display high levels of sphingolipids and sterol and lower levels of PC and PI. Thus, in the present study, the relative increase of FST and SPG and decrease of PC and PI in Seine vs. Douarnenez could be linked to an increase of the density of lipid rafts in the endoplasmic reticulum, to improve the cell capacity for xenobiotic metabolism.

The endoplasmic reticulum is a dynamic organelle that can proliferate in response to xenobiotic exposure. The specific lipid composition of rafts allows for more xenobiotics and endogenous substrate metabolism by increased binding efficiency between CYP1A2 and CPR (NADPH-cytochrome P450 reductase) [81]. Membrane phospholipids seem to accelerate the enzymatic activity of cytochrome P4501A by changing its structural conformation, thus controlling the detoxification of xenobiotics [82]. Complex sphingolipids associated with sterols are essential for maintaining endoplasmic reticulum homeostasis, and perturbation in sphingolipid levels activates an endoplasmic reticulum stress-mediated to propagate apoptotic signals to the mitochondria [83].

### 4.5. Potential Relationships between Phospholipids, Xenobiotic Metabolism, Antioxidant Defense and Bioenergetics in Contrasted Environments

Flounder in post-spawning stage display the lowest condition index and muscle reserve [28] characterizing exhausted fish. Thus, in the present study, also conducted on post-spawning fish, the negative correlation between CCO activity and protein concentration in muscle in the two systems (Seine and Douarnenez) could indicate two contrasted physiologies; fish displaying a higher metabolism and reduced reserves vs. fish showing a reduced metabolism but maintaining higher reserves. These opposite bioenergetic patterns were also described in natural fish populations [20,84], confirming that the phenotypic diversity in key metabolic traits can lead to differential plasticity towards environmental change [85].

The Douarnenez fish were weakly contaminated by organic pollutants and showed a significant metal contamination mainly by Pb and Zn. They displayed a positive correlation between liver G6PDH and brain AChE activities, condition index, muscle protein, and PS (phosphatidylserine), with this group of markers being negatively correlated with muscle CCO activity. We suggest that individuals displaying the better capacity to manage the oxidative stress are able to cope with the metal stress, maintaining a reduced neurotoxicity and higher muscle reserves. The PS is an anionic phospholipid that provides negatively charged environment that should facilitate protein insertion in membrane, and thus should play a key role in regulation of cellular functions of the plasma membrane as well as intracellular organelles [81]. The Douarnenez fish also displayed a positive correlation between SPG and liver EROD activity. In the context of a weak pollution by organic pollutants, it seems that the increase of sphingolipids particularly located in endoplasmic reticulum could increase the catalytic activity of the cytochrome P4501A, as seen previously [81,82].

The Seine fish were exposed to a complex mixture of organic and metallic pollutants in a heavily contaminated estuary. They displayed different relationships between markers relatively to Douarnenez fish. In the Seine population, the AChE activity was negatively correlated with PS and FST (free sterol), with these two membrane lipids displaying higher levels in Seine vs. Douarnenez. Thus, the potential relationship between neurotoxicity and membrane lipids could be dependent on the nature of the chemical stress.

Furthermore in the Seine population, a positive correlation was detected between three markers: G6PDH and CCO activities, and SPG. Recent studies underlined that bioactive lipids and sphingolipids in particular are functionally defined as lipid species, levels of which respond to the action of specific stimuli [86]. Thus, bioactive lipids are component of signalling networks which distinguishes them from other lipids that have structural and/or energetic functions. The functions now attributed to bioactive sphingolipids encompass all major aspects of cell biology (e.g., cell growth, cell cycle, cell death, cell senescence, inflammation, immune response, metabolism, stress response) [86]. Reduction in complex sphingolipid levels causes loss of viability, most likely due to the induction of mitochondria-dependent apoptotic cell death pathway, accompanied by changes in mitochondrial and endoplasmic reticulum morphology and endoplasmic reticulum stress [83]. Thus, in the heavily polluted Seine estuary, we suggest that the fish displaying the higher sphingolipid levels are the more efficient to maintain their mitochondria integrity and consequently the higher levels of CCO activity. We hypothesise that the high activity of the Cytochrome C Oxidase accelerates electron flow into complex III of the oxidative phosphorylation chain, preventing ROS production in these organelles [87,88], thus allowing a higher G6PDH activity. All these links suggested between phospholipids, xenobiotic metabolism, antioxidant defence, and bioenergetics must indeed be considered cautiously because our assumptions are only based on correlations between markers.

### 4.6. Genetic Variability in the Two Populations

Considering neutral genetic markers (microsatellites), the intra-population genetic variability was similar in the Seine and Douarnenez populations. Thus, the present study showed no loss in genetic diversity in the heavily polluted Seine population compared with the moderately contaminated Douarnenez population. We suggest that the current maintenance of the genetic diversity in the Seine could be linked to the absence of frequent demographic bottlenecks in this population, and thus to a limited possibility of genetic drift or inbreeding leading to a loss of genetic diversity. Conversely, severe inbreeding and small effective population sizes have been shown to impact seriously the genetic variability of winter flounder populations collected in chronically contaminated bays and estuaries over Long Island, New York [29].

In the present study, no genetic divergence was observed between the two populations, suggesting that the Douarnenez population is genetically very close to other flounder populations located in the English Channel (including the Seine population). We suggest that (1) a recent divergence of populations combined with large effective population sizes and/or (2) gene flow between flounder populations over the Channel, may explain the reduced genetic structure in this area, considering neutral genetic markers. However, selective pressures related to contaminants are acting on the Seine flounder population which is clearly differentiated from less polluted populations, considering the polymorphism of genes involved in energy metabolism, apoptosis, and detoxification [22-24).

## 5. Conclusions

This study confirmed the pertinence of the European flounder (*Platichthys flesus)* to assess the environmental status of coastal zones and estuaries. Lipid reserves were seriously depleted in heavily contaminated fish of the Seine estuary vs. those from the moderately contaminated bay of Douarnenez. The integration of the main markers in heavily vs. moderately contaminated fish allowed to hypothesise a relationship between the levels of sphingomyelin—phosphatidylserine—free sterols, and the fish capacity to manage the oxidative stress and to metabolize xenobiotics. Finally, we suggest that phospholipid metabolism changes could be relevant markers in ecotoxicology [89,90]. Specifically, their combination with biomarkers of energy metabolism and oxidative stress could elucidate the pathways and mechanisms in xenobiotic acclimation and resilience.

Concerning the ecological status of the heavily contaminated flounder population of the Seine, we hypothesise that this population was not affected by successive demographic bottelenecks and thus has maintained its neutral genetic variability until nowadays. However, the physiological cost of the local adaptation of this flounder population to the chemical stress was identified in the past by fish fitness loss (reduced condition index and growth rate, weak fecundity) [10,91], and was confirmed in the present study by the increased cost of living (reduced lipid reserve, high metabolic rate). Furthermore a reduced thermal plasticity was highlighted in the Seine flounder population compared to a less polluted population [21], as in another fish population showing a pollution adaptation in North America [92]. Finally, we suggest that the high physiological cost of tolerance to toxicants in the Seine flounder population could compromise its capacity to respond in the future to an additional stressor like warming waters in shallow depth. Thus, this population could be submitted to an ecological risk.

## Figures and Tables

**Table 1:** Determination of elemental concentrations (in µg.g^−1^, dry material), compared to certified values for reference materials from NRCC: DORM-4 (fish protein), DOLT-5 (dogfish liver), and TORT-3 (lobster hepatopancreas).

	DORM-4		DOLT-5		TORT-3	
	Measured (*n* = 5)	Certified	Measured (*n* = 5)	Certified	Measured (*n* = 5)	Certified
As	7.6 ± 0.7	6.9 ± 0.5	32 ± 6	35 ± 6	59 ± 11	60 ± 4
Cd	0.31 ± 0.04	0.30 ± 0.02	12 ± 2	14.5 ± 0.6	34 ± 5	42 ± 2
Co	0.28 ± 0.03	0.25 *	0.29 ± 0.09	0.27 ± 0.03	1.04 ± 0.06	1.06 *
Cr	1.5 ± 0.1	1.9 ± 0.2	1.0 ± 0.2	2.3 ± 0.6	0.9 ± 0.1	1.9 ± 0.3
Cu	16.1 ± 0.9	15.7 ± 0.5	31 ± 4	35 ± 3	350 ± 80	500 ± 30
Ni	1.20 ± 0.08	1.3 ± 0.2	0.7 ± 0.2	1.7 ± 0.7	4.2 ± 0.4	5.3 ± 0.3
Pb	0.30 ± 0.09	0.42 ± 0.06	0.04 ± 0.03	0.16 ± 0.04	0.26 ± 0.07	0.23 ± 0.02
V	1.5 ± 0.1	1.6 ± 0.2	0.50 ± 0.04	0.51 ± 0.06	8.8 ± 0.3	9.1 ± 0.4
Zn	49 ± 7	52 ± 3	96 ± 14	105 ± 6	125 ± 9	136 ± 6

* indicative value.

**Table 2:** PAH and PCBs concentrations in liver (L) and muscle (M) in Seine estuary (S) and Douarnenez bay (D). (one sample: a pool of five fish tissues).

ng.g^−1^ DW	LS	LD	MS	MD
Naphthalene	97.26	-	-	-
PCB 101	91.32	-	70.12	-
PCB 118	57.56	24.96	69.85	-
PCB 153	255.20	-	164.93	-
PCB 138	290.65	45.49	167.34	-
PCB 180	26.20	-	-	-

**Table 3:** Muscle lipid content expressed as µg of lipid per milligram of fresh weight (storage lipids and membrane lipids) in Douarnenez (DZ) vs. Seine (SE) fish. STEST: sterol esters, GLETH: glyceride ethers, TG: triacylglycerols, FFA: free fatty acid, ALC: fatty alcohols, FST: free sterols, SPG: sphingomyelin, LPC: lysophosphatidylcholine, PC: phosphatidylcholine, PS: phosphatidylserine, PI: phosphatidylinositol, CL: cardiolipins, PE: phosphatidylethanolamine. Sum of storage lipids (Ʃ SL), membrane lipids (Ʃ ML) and total lipids (ƩALL). (lipid mean; sd = standard deviation; significant difference between Douarnenez and Seine: ** *p*-value < 0.01, *** *p*-value < 0.001; NS no significant).

Location	DZ			SE	
	mean	sd	sign.	mean	sd
Storage lipids					
ST EST	0.02	0.03	***	0.06	0.05
GL ETH	0.01	0.02	NS	0.02	0.03
TG	1.96	1.82	**	1.01	1.21
FFA	0.05	0.05	NS	0.04	0.03
ALC	0.04	0.12	NS	0.02	0.03
Membrane lipids					
FST	0.40	0.09	**	0.45	0.10
SPG	0.20	0.12	***	0.35	0.10
LPC	0.19	0.08	***	0.10	0.08
PC	5.40	0.71	***	4.42	0.68
PS	0.27	0.05	**	0.32	0.10
PI	0.87	0.15	***	0.64	0.12
CL	0.33	0.15	NS	0.27	0.12
PE	2.47	0.39	***	2.17	0.31
∑ SL	2.08		**	1.15	
∑ ML	10.14		**	8.73	
∑ ALL	12.21		**	9.88	

**Table 4:** Genetic diversity: allelic diversity (Na: Number of alleles), observed heterozygosity (Ho), expected heterozygosity (He) in the Douarnenez bay and the Seine estuary.

Locus	Panels	Douarnenez			Seine		
		N_a_	H_o_	H_e_	N_a_	H_o_	H_e_
Nplaf_8	1	4	0.444	0.448	4	0pl.300	0.339
Nplaf_14	3	3	0.378	0.413	4	0.352	0.342
Nplaf_15	2	5	0.272	0.338	5	0.718	0.569
Nplaf_23	2	5	0.500	0.637	6	0.419	0.580
Nplaf_24	3	6	0.588	0.645	6	0.656	0.664
Nplaf_25	3	6	0.756	0.673	4	0.685	0.658
Nplaf_28	3	7	0.620	0.728	6	0.650	0.710
Nplaf_30	3	4	0.166	0.205	4	0.437	0.371
Nplaf_35	1	17	0.971	0.917	19	0.903	0.915
PFUSC3	1	6	0.529	0.478	3	0.382	0.349
PFUSC4	3	3	0.527	0.526	6	0.666	0.568
PFUSC7	2	10	0.617	0.742	7	0.709	0.721
PFUSC8	1	4	0.450	0.502	3	0.428	0.438
PL142	2	14	0.896	0.878	11	0.619	0.833
StPfl001	1	21	0.742	0.832	18	0.774	0.868
StPfl002	3	10	0.638	0.649	6	0.558	0.626
StPfl003	2	5	0.756	0.679	4	0.454	0.543
StPfl005	2	3	0.457	0.452	4	0.457	0.530
StPfl015	1	9	0.432	0.747	9	0.363	0.604
StPfl025	2	7	0.500	0.551	7	0.687	0.617
Multilocus		7.45	0.562	0.602	6.8	0.561	0.592
